# Supplementary material for: JacLy: a Jacobian-based method for the inference of metabolic interactions from the covariance of steady-state metabolome data
Source: PeerJ. 2018 Dec 6;6:e6034. doi: 10.7717/peerj.6034 (PMC6286809; doi:10.7717/peerj.6034)
Supplement: Figure S1 [file peerj-06-6034-s003.pdf]

Supplemental Figure S1

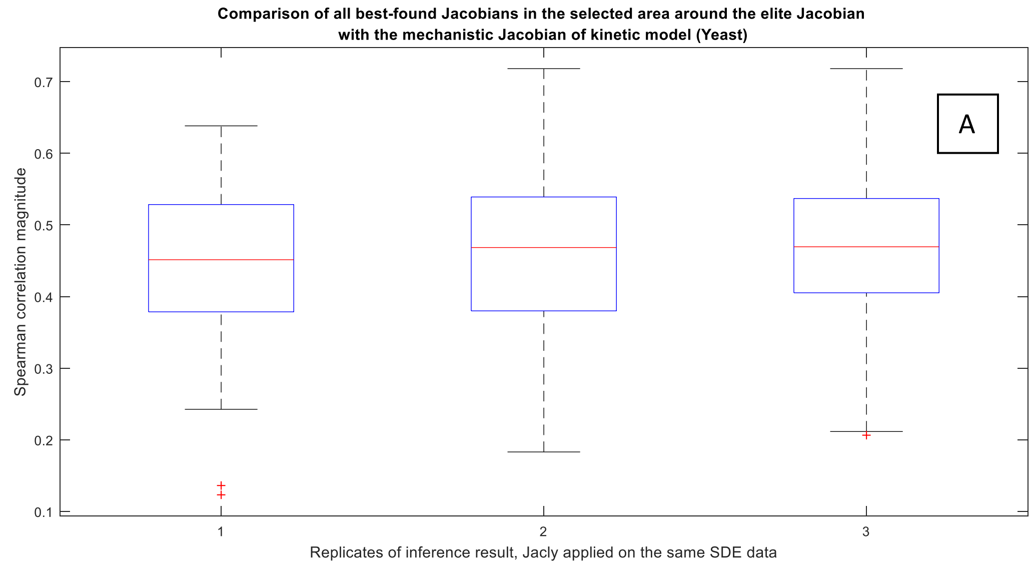

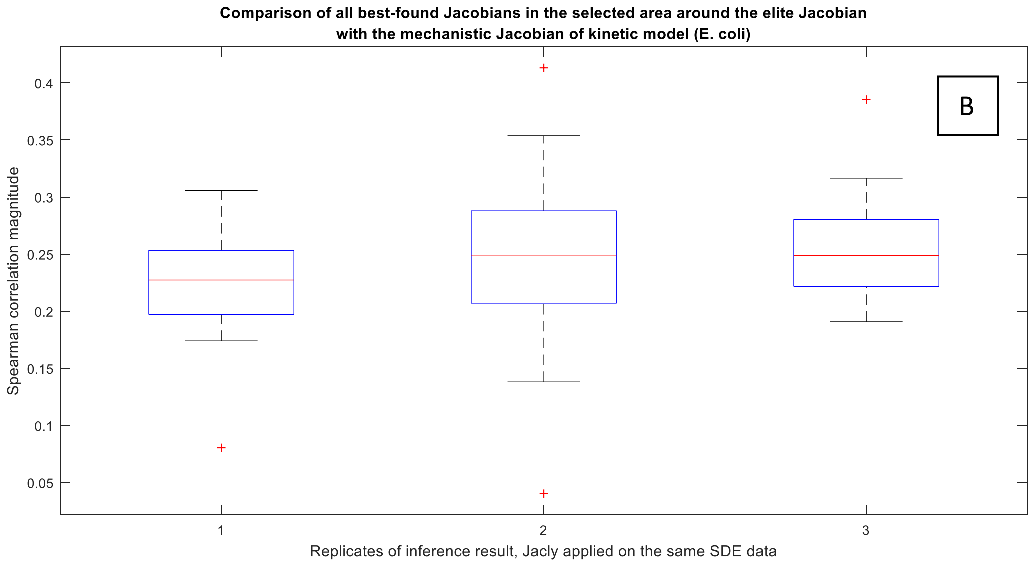

Figure S1: The Spearman correlation between the predicted Jacobian matrix values by JacLy and the calculated values from the kinetic models are shown here for both Yeast and E.coli. JacLy was applied three times (due to its stochastic nature) for each organism, and the correlation was calculated for each of the best-Jacobians determined around the elite Jacobians. The results are given below in the form of boxplots. (a) The results for the yeast. 1: 60 best-found Jacobians, 2: 100 best-found Jacobians, 3: 96 best-found Jacobians. (b) The results for *E. coli*. 1: 12 best-found Jacobians, 2: 100 best-found Jacobians, 3: 11 best-found Jacobians.
